# Chromatography related performance of the Monitor for AeRosols and GAses in ambient air (MARGA): laboratory and field-based evaluation

**DOI:** 10.5194/amt-10-3893-2017

**Published:** 2017-10-24

**Authors:** Xi Chen, John T. Walker, Chris Geron

**Affiliations:** National Risk Management Research Laboratory, Office of Research and Development, US Environmental Protection Agency, Research Triangle Park, North Carolina, 27711, USA

## Abstract

Evaluation of the semi-continuous Monitor for AeRosols and GAses in ambient air (MARGA, Metrohm Ap-plikon B.V.) was conducted with an emphasis on examination of accuracy and precision associated with processing of chromatograms. Using laboratory standards and atmospheric measurements, analytical accuracy, precision and method detection limits derived using the commercial MARGA software were compared to an alternative chromatography procedure consisting of a custom Java script to reformat raw MARGA conductivity data and Chromeleon (Thermo Scientific Dionex) software for peak integration. Our analysis revealed issues with accuracy and precision resulting from misidentification and misintegration of chromatograph peaks by the MARGA automated software as well as a systematic bias at low concentrations for anions. Reprocessing and calibration of raw MARGA data using the alternative chromatography method lowered method detection limits and re-duced variability (precision) between parallel sampler boxes. Instrument performance was further evaluated during a 1-month intensive field campaign in the fall of 2014, including analysis of diurnal patterns of gaseous and particulate water-soluble species (NH_3_, SO_2_, HNO_3_, $NH_{4}^{+}$, $SO_{4}^{2-}$ and $NO_{3}^{-}$^,^ gas-to-particle partitioning and particle neutralization state. At ambient concentrations below ~ 1 µg m^–3^, concentrations determined using the MARGA software are biased +30 and +10 % for $NO_{3}^{-}$ and $SO_{4}^{2-}$, respectively, compared to concentrations determined using the alternative chromatography procedure. Differences between the two methods increase at lower concentrations. We demonstrate that positively biased $NO_{3}^{-}$ and $SO_{4}^{2-}$ measurements result in overestimation of aerosol acidity and introduce nontrivial errors to ion balances of inorganic aerosol. Though the source of the bias is uncertain, it is not corrected by the MARGA online single-point internal LiBr standard. Our results show that calibra-tion and verification of instrument accuracy by multilevel external standards is required to adequately control analytical accuracy. During the field intensive, the MARGA was able to capture rapid compositional changes in PM_2.5_ due to changes in meteorology and air mass history relative to known source regions of PM precursors, including a fine $NO_{3}^{-}$ aerosol event associated with intrusion of Arctic air into the southeastern US.

## 1 Introduction

Secondary inorganic aerosols are formed from gaseous precursors including ammonia (NH_3_), nitric acid (HNO_3_) and sulfur dioxide (SO_2_), producing ammonium nitrate (NH_4_NO_3_), ammonium bisulfate (NH_4_HSO_4_) and ammonium sulfate ((NH_4_)_2_SO_4_) particles. These gaseous precursors and particulate matter, which partition between phases to establish a thermodynamic equilibrium of ammonium–sulfate–nitrate (Finlayson-Pitts and Pitts, 2000; Seinfeld and Pandis, 2006), represent a significant fraction of PM_2.5_ (Seinfeld and Pandis, 2006; Pinder et al., 2007) and contribute to atmospheric deposition of nutrients and acidity. The implementation of National Ambient Air Quality Standards has reduced emissions of NO_*x*_ and SO_2_; however, NH_3_ is not regulated and has not been routinely monitored until relatively recently (Puchalski et al., 2015). Nevertheless, to further reduce fine particulate matter, controlling NH_3_ emissions has been suggested to be more cost-effective than further reductions of NO_*x*_ and SO_2_ in some cases (Vayenas et al., 2005; Pinder et al., 2007). Reduction of NH_3_ emis-sions may also represent the most effective strategy for reducing atmospheric nitrogen deposition to acceptable levels (Li et al., 2016) in some ecosystems. High-frequency simultaneous measurements of the gas and aerosol components of the ammonium–sulfate–nitrate system are required to investigate inorganic aerosol characteristics (e.g., phase partitioning, acidity) and formation processes and to quantify the dry component of nitrogen deposition.

Traditionally, integrated denuder and/or filter-based techniques (i.e., 24 h or longer) have been used to monitor inorganic aerosols and their precursors (Trebs et al., 2004, and references therein; Benedict et al., 2013; Chen et al., 2014). The disadvantages of poor temporal resolution and labor intensity, as well as positive and negative sampling artifacts, make these methods difficult to deploy for extended periods of time and of limited use for characterization of rapidly changing atmospheric conditions. Recent development of near-real-time semi-continuous analyzers, including the particle-into-liquid sampler (PILS-IC, Metrohm AG, Herisau, Switzerland), particle collector–ion chromatograph (PC-IC), aerosol mass spectrometer (AMS, Aerodyne Research Inc., USA), ambient ion monitor–ion chromatograph (AIM-IC, URG Corp. and Dionex Inc., USA) and the Monitor for AeRosols and GAses in ambient air (MARGA, Metrohm Applikon B.V., the Netherlands), facilitate monitoring inorganic atmospheric constituents with much higher time resolution (Jayne et al., 2000; Weber et al., 2001; Al-Horr et al., 2003; Trebs et al., 2004; Schaap et al., 2011; Markovic et al., 2012). A version of the MARGA incorpo-rating two sample boxes (MARGA 2S), similar to the system described here, has recently been used to quantify dry deposition using a micrometeorological flux gradient method (Rumsey and Walker, 2016).

MARGA’s capability of near-real-time (hourly) simultaneous measurement of water-soluble particulate species as well as their gaseous precursors makes it a state-of-art research instrument. Such time-resolved measurements allow investigation of highly time-sensitive, rapidly changing pollution episodes as well as aerosol processes such as gas–particle partitioning and neutralization state. The MARGA has been deployed in widely varying environments to monitor ambient gaseous and particulate water-soluble species including NH_3_, SO_2_, HNO_3_, $NH_{4}^{+}$, $SO_{4}^{2-}$ and $NO_{3}^{-}$ (Schaap et al., 2011; U.S. EPA, 2011; Makkonen et al., 2012; Mensah et al., 2012; Khezri et al., 2013; Huang et al., 2013; Rumsey et al., 2014; Shi et al., 2014; Allen et al., 2015; Twigg et al., 2015; Rumsey and Walker, 2016). Although the MARGA denuder and steam jet aerosol collector (SJAC) have been evaluated for collection efficiency of gases and particles (Wyers et al., 1993; Khlystov et al., 1995), there is relatively limited data on accuracy and precision of concentration measurements (Weber et al., 2003; Trebs et al., 2004; Makkonen et al., 2012; Lee et al., 2013; Rumsey et al., 2014; Allen et al., 2015). Phillips et al. (2013) found that HNO_3_ determined by the MARGA’s wet rotating denuder (WRD) displays a cross-sensitivity to N_2_O_5_. The magnitude of the resulting positive bias in HNO_3_ is highly dependent on the ambient conditions (eg. NO_*x*_, O_3_, biogenic VOC concentrations and temperature) responsible for N_2_O_5_ production. Lee et al. (2013) observed differences in $SO_{4}^{2-}$, $NH_{4}^{+}$ and $NO_{3}^{-}$ at a suburban site in Hong Kong, where an AMS instrument measured only 33–60 % of the PM mass measured by a collocated MARGA. Part of the difference was attributed to different particle size cut of the inlets used (PM_1.0_ for AMS and PM_2.5_ for MARGA). Rumsey et al. (2014) compared the MARGA to a reference time-integrated denuder/filter pack system. SO_2_, $SO_{4}^{2-}$ and $NH_{4}^{+}$ agreed within 15 % between the two systems; however, HNO_3_ and NH_3_ comparisons showed an underestimation by MARGA of 30 %, mostly likely due to loss to the surface of the long (≈ 4 m) polyethylene sample tubing used. Though differences between the MARGA and other measurement systems have been observed, the extent to which the differences may be attributable solely to chromatography has not been evaluated.

The objective of this study is to evaluate MARGA performance with a focus on accuracy and precision characteristics related to automated chromatography analysis. Specifically, we investigate misidentification and misintegration by the MARGA software as well as errors and uncertainties resulting from such issues. To aid efficiency and flexibility in the reprocessing of MARGA chromatograms, an alternative chromatography procedure, based on offline analysis of raw MARGA data, was employed. Using laboratory standards, analytical accuracy, precision and method detection limits derived from the two chromatograph processing methods were compared. Field measurements were used to further evaluate instrument performance and to demonstrate the ability of the MARGA instrument to resolve important atmospheric processes, including diurnal patterns of observed gaseous (NH_3_, SO_2_, HNO_3_) and particulate water-soluble species ($NH_{4}^{+}$, $SO_{4}^{2-}$ and $NO_{3}^{-}$), fine particle neutralization state and changes in atmospheric composition related to synoptic meteorological patterns. Using aerosol neutralization state as a case study, the impact of chromatography errors on measurement accuracy was assessed.

## 2 Methods and materials

### 2.1 MARGA system

Details and principles of the MARGA system have been previously described (Rumsey et al., 2014; Rumsey and Walker, 2016). Briefly, the MARGA sampler box consists of a WRD and a SJAC, which enables semi-continuous collection and measurement of gaseous and water-soluble inorganic particulate species in the ambient air. When drawn through the WRD, gaseous species are collected by diffusion into a liquid film while particles pass through the WRD to the SJAC, where supersaturation grows the particles by condensation. Liquid samples from the WRD and SJAC are continuously collected in individual syringes and analyzed by ion chromatography (IC) on an hourly basis at the detector unit. Cation and anion sample loop volumes are 500 and 250 µL, respectively. By employing two sets of liquid syringes, a set of samples is collected while samples from the previous hour are analyzed. To monitor accuracy and automatically adjust concentrations, liquid samples are mixed with an internal lithium bromide (LiBr) standard at a fixed ratio before injection for IC analysis.

### 2.2 Chemical materials

DI water (18.2 MΩ cm, Milli-Q Reference system, Millipore) with 10 ppm H_2_O_2_ (30 % certified ACS grade, Fisher Scientific) was used as absorbance solution for the MARGA WRD and SJAC sample collection. H_2_O_2_ was added to prevent bacteria growth and subsequent loss of $NH_{4}^{+}$ The MARGA internal standard LiBr (> 99 %, ACROS Organics) aqueous solution was prepared at concentrations of 320 µg L^−1^ Li^+^ and 3680 µg L^−1^ Br^–^ Solid chemical standards NH_4_NO_3_, NH_4_Cl, (NH_4_)_2_SO_4_, NaNO_3_, KCl, CaCl_2_ 2H_2_O and MgSO_4_ 7H_2_O ( 99 % certified ACS grade, Fisher Scientific) were used to prepare stocks and various levels of liquid external standards. Certified aqueous analytical standard solutions purchased from Alltech Associates (Anion Mix 1, Cation Mix B, Alltech Associates, Inc.) served as accuracy check standards. We note here that “internal” standard refers to the MARGA LiBr standard that is mixed with every MARGA liquid sample immediately up-stream of the IC injection loop. “External” standards refer to liquid standards that are introduced at the WRD and SJAC, as described in more detail below.

### 2.3 Chromatography

MARGA proprietary chromatography software consists of an online version used for automated analysis when the instrument is in measurement mode and a “MARGA tool”, so named by the manufacturer, used for offline analysis of chromatograms, either individually or in batches, but otherwise identical to the online version. In both cases, liquid analyte concentrations are determined by calculating the total amount of injected sample directly from the conductivity measurement following the method of van Os et al. (1984). As mentioned previously, accuracy is controlled by adjusting the measured concentration based on a single-point internal LiBr standard, at a working concentration of 320 µg L^−1^ of Li^+^ and 3680 µg L^−1^ of Br, which is injected with each sample. The MARGA software does not employ a multipoint calibration curve.

During post-processing of field data, it was discovered that peaks integrated by the MARGA tool showed a certain degree of misidentification and inconsistent integration. Specific integration issues include incorrectly defined baseline due to peak fronting and tailing and shifting between “drop perpendicular” and “valley to valley” integration options among samples (shown in Supplement). As indicated by the examples shown in Supplement, baseline selection by the MARGA tool could vary from sample run to run, which could introduce significant errors and uncertainties. Integration issues are particularly problematic when the IC analytical columns deteriorate due to extended use. Under such conditions, unresolved peaks occurred more frequently.

In addition to misidentification and misintegration issues with the MARGA software, reintegration of individual peaks with the MARGA tool was found to be inefficient and inflexible. Although the MARGA tool contains adjustable integration parameter settings such as peak search sensitivity and peak search smoothing, the parameters are applied to all chromatograms. For example, the adjusted parameter may achieve the desired integration for a particular misintegrated peak, but other peaks which were deemed as integrated properly prior to any adjustments may subsequently be improperly integrated. The inability to manually adjust the integration for individual peaks makes post-processing of chromatograms time consuming. Hence, an alternative chromatography software (Chromeleon V7.2, Thermo Scientific Dionex) was tested for reprocessing of MARGA chromatograms.

In order to import MARGA generated chromatograms to the Chromeleon chromatography data processing system, raw MARGA chromatography data (dat format) were converted to time series of conductivity (txt format) using the MARGA tool. Using the Chromeleon generated template (cdf format) file, as well as a custom Java script, a batch of MARGA conductivity time series (txt format) files is converted to their corresponding cdf format. A folder of conductivity data files in cdf format is then imported to Chromeleon for chromatogram reprocessing.

MARGA and Chromeleon approaches were compared in terms of peak areas and calculated concentrations of internal and external liquid standards, as well as determinations of laboratory blanks, method detection limits and air concentrations during ambient sampling. To compare integration characteristics between the MARGA tool and Chromeleon software, a series of external liquid standards (Table S1 in the Supplement), representing a range of concentrations equivalent to ≈ 0.05–0.5 µg m^−3^ $NH_{4}^{+}$, $NO_{3}^{-}$ and $SO_{4}^{2-}$ in air, was run through the MARGA instrument with the air pumps and SJAC steam generator disconnected. This configuration allowed liquid standards to pass through the entire sampling (i.e., WRD and SJAC and liquid sampling lines) and analytical (i.e., syringes and ICs) components of the system. The resulting chromatograms were used to generate a calibration curve using Chromeleon, in which peak areas were related to liquid standard concentration (µg L^−1^). These peak areas and concentrations were then compared directly to peak areas and concentrations generated by the MARGA software (without any further manual peak integration adjustment), the latter being adjusted only by the internal LiBr standard. A certified accuracy check standard was used to evaluate the accuracy of the calibration curves generated by Chromeleon and all of the analytes were found to be within the 10 % accuracy check criteria. System blanks using absorbance solution were evaluated in the same manner as the external liquid standards. Finally, both the MARGA internal standard (LiBr) and a subset of the external standards were verified by independent analysis on a Dionex ICS-2100 (Thermo Scientific, Waltham, MA) multipoint calibrated with additional certified standards. Due to different loop size and corresponding detection limit of the Dionex system, only a subset of the external standards was independently verified.

### 2.4 Field study

Field measurements were conducted in a grass field at the Blackwood Division of Duke Forest (35.98^°^ N, 79.09^°^ W) near Chapel Hill, NC. Duplicate MARGA sample boxes (SB1 and SB2) were positioned in parallel (i.e., collocated) with inlets ≈ 1.5 m above the ground. Both MARGA sample boxes employed a Teflon-coated cyclone-type inlet with an aerodynamic 2.5 µm cut size at a flow rate of 16.7 LPM (URG-2000–30EH, University Research Glassware Corporation). A short (0.2 m) length of 25.4 mm o.d. Teflon tubing connected the atmospheric inlet to the MARGA denuder. MARGA sampler and detector boxes were equipped with weather protection enclosures which were temperature controlled at 25^°^C.

Sampler air flow rates were measured and verified weekly by connecting a NIST traceable primary standard flow meter (Bios DryCal DC-Lite flowmeter, Mesa Laboratories, Inc., Lakewood, CO) to the sampler inlets. Based on the calibration by the flow meter, MARGA reported flow rates were overestimated by 6 and 8 % for sample box 1 (SB1) and 2 (SB2), respectively, and air concentrations were adjusted accordingly. Initial data validation was conducted by monitoring the MARGA automated status codes; data with internal standard LiBr responses outside of ±10 % nominal concentrations were invalidated and excluded from further analysis.

To compare air concentrations derived from MARGA and Chromeleon software, the liquid calibration curves (see above Sect. 2.2) generated by Chromeleon were used to calculate liquid concentrations and, by combining with air and liquid flow rates, corresponding air concentrations were derived. The Chromeleon-derived air concentrations were then compared to air concentrations generated by the MARGA software, which used only the internal LiBr standard as a calibration adjustment. For this comparison, the same air and liquid flow rates were used. Both sets of air concentrations were corrected for system blanks and air flow rate calibrations. The MARGA was operated continuously in the field from 15 October to 17 November 2014. However, due to a failure of the IC degasser unit, no valid data were generated from 31 October to 2 November 2014.

### 2.5 Ancillary field data

A 10 m meteorological station is maintained and managed at Duke Forest by the North Carolina Division of Forest Resources and Bureau of Land management. Verified hourly metrological data were obtained online (http://mesowest.utah.edu). Concentrations of PM_2.5_ mass (TEOM model 1400ab, R&P Thermo Scientific, Franklin, MA) and organic/elemental carbon (OC/EC, model 4 semi-continuous field analyzer, Sunset Laboratory, Inc., Hillsborough, NC) were measured adjacent to the MARGA instrument. Backward air mass trajectories were calculated for select periods using the Hybrid Single Particle Lagrangian Integrated Trajectory (HYSPLIT) model (Draxler and Rolph, 2003) with NOAA ARL EDAS 40 km meteorological data. Trajectories were run for 168 h periods at an arriving height of 500 m above the ground level. To aid interpretation of the back trajectories, facility emission inventory data for NO_x_, SO_2_ and NH_3_ were retrieved from the 2011 National Emission Inventory database (http://www.epa.gov/ttn/chief/ net/2011inventory.html).

## 3 Results and discussion

### 3.1 Laboratory study of chromatography characteristics

MARGA chromatograms were systematically examined by running a series of liquid external standards over a range of concentrations listed in Table S1. Each standard level was analyzed for approximately 20 h, producing *N* = 80 observations for four analytical channels combined (two sample boxes for gas and aerosol channels). The same sets of chromatograms were reprocessed by Chromeleon to generate multipoint calibration curves for each analyte. Peaks that were obviously misintegrated by the MARGA tool were not included in this analysis. Relationships between peak area and standard concentration were linear except for $SO_{4}^{2-}$, for which a polynomial fit was adopted to better represent the entire concentration range. All calibration curves had *r*^*2*^ values >0.999. A certified check standard was used to evaluate the accuracy of the calibration curves generated by Chromeleon and all analytes were found to be within the 10 % accuracy check criteria. Using absorbance solution to assess contamination, blank concentrations of $NO_{3}^{-}$ and $SO_{4}^{2-}$ reported by Chromeleon were 0.002 and 0.080 µg m^−3^, respectively, while the corresponding system blanks determined by the MARGA tool were 0.018 and 0.109 µg m^−3^. $NH_{4}^{+}$ was not detectable in the blank solution.

Table 1 lists estimated method detection limits for the species of interest calculated using both the MARGA tool and Chromeleon. Method detection limits were calculated as 2.58 standard deviation of the lowest detectable external standards, a statistical method described in detail by Currie (1999). Method detection limits calculated using the MARGA software are substantially larger than corresponding detection limits calculated with Chromeleon, indicating more variability in the MARGA integrations from sample to sample. Such inconsistency will translate to larger uncertainties for low concentration samples. This is particularly important when attempting to resolve very small differences between two MARGA sample boxes, a requirement for flux gradient applications (Rumsey and Walker, 2016). Error propagations inherited from misintegration could be minimized by reexamining the chromatograms. We note that the detection limits of the instrument evaluated here are larger, particularly for anions, than those reported by Rumsey and Walker (2016), which used the same MARGA software but a different instrument. This indication of variability demonstrates the need to characterize individual measurement systems. The detection limits calculated with Chromeleon are more similar to those reported by Rumsey and Walker (2016).

Table S2 lists the internal standard peak areas as integrated by the MARGA tool and Chromeleon for each of the corresponding external standard levels. Note that while the concentrations of anions and cations in the external standards vary by level, the actual concentration of the internal standard does not. For both Li^+^ and Br^–^, systematically larger peak areas are calculated by the MARGA software. While the systematic difference for Br is rather consistent (17 %), differences in Li^+^ between the two software techniques decrease with increasing external standard concentration. As the peak areas of Na^+^ and $NH_{4}^{+}$ increase, the close retention times of Na^+^, $NH_{4}^{+}$ and Li^+^ cause the peaks to appear more like unresolved lumps (i.e., peak merging effect). At these higher standard concentrations, the MARGA software underestimates the Li^C^ peak area relative to Chromeleon and integration from sample to sample becomes less consistent. This is likely due to the MARGA software frequently shifting between “drop perpendicular” and “valley to valley” integration options between samples, introducing more variability to the calculated areas (see Supplement Fig. S1). For consistency, the drop perpendicular integration option was adopted for all Chromeleon reprocessing. We observed that as the concentration levels increase, the errors due to adopting different integration options could be as much as 6 % at the highest external standard concentration equivalent to ≈ 10.5 µg m^−3^. In summary, the consistent 17 % difference in Br peak areas between software packages is not necessarily a source of error in the final calculation of MARGA liquid concentrations. For Li^+^, the variability in integration and decrease in the difference in peak area between the two software packages at higher standard levels would translate to systematic differences in corresponding $NH_{4}^{+}$ liquid concentrations above 100 µg L^+^ (≈ 2.5 µg m^−3^ in air).

In addition to underestimation of Li^+^, other issues associated with MARGA processing of cation chromatograms include misidentification of $NH_{4}^{+}$ as Na^+^ when a negligible Na^+^ peak existed and misidentification of $NH_{4}^{+}$ and Na^+^ peaks together as a single $NH_{4}^{+}$ peak. For anion chromatograms, $NO_{3}^{-}$ peaks were rather frequently discovered as not identified at all; $SO_{4}^{2-}$ peaks were found to have an incorrectly defined baseline due to peak fronting and tailing (see Figs. S2–S5). These issues become more prevalent with column age.

### 3.2 Field study

In order to assess the potential impact of chromatography related analytical errors observed during the laboratory evaluation, MARGA performance was further investigated during a 1-month field campaign. Air concentrations generated by the MARGA tool and Chromeleon are compared over a range of chemical and meteorological conditions, using particle neutralization state as a case study. Intrusion of Arctic air into the southeastern US provided an opportunity to observe rapidly changing and distinct patterns of gas-to-particle partitioning within the ammonium–nitrate–sulfate system. In the following sections, air concentrations presented in time series and summary statistics describing ambient measurements were generated by Chromeleon unless otherwise indicated.

#### 3.2.1 MARGA accuracy

Chromatograms reprocessed by the MARGA tool were individually examined and concentrations were filtered for periods of instrument malfunction, peak misintegration and LiBr internal standard outside ±10 % of the nominal target concentration. This filtering procedure would include low concentrations in which there was an obvious problem with the original peak integration. Filtered data were not included in the comparison between the MARGA tool and Chromeleon. Table S3 presents the percentage of data excluded from the comparison. $NO_{3}^{-}$ peaks appeared to be the analyte most affected, especially in the case of HNO_3_ (up to 6.2 % of the data). At sampling sites where HNO_3_ concentrations are typically below 1 µg m^−3^, data rejection may be more extensive.

**Figure 1.**
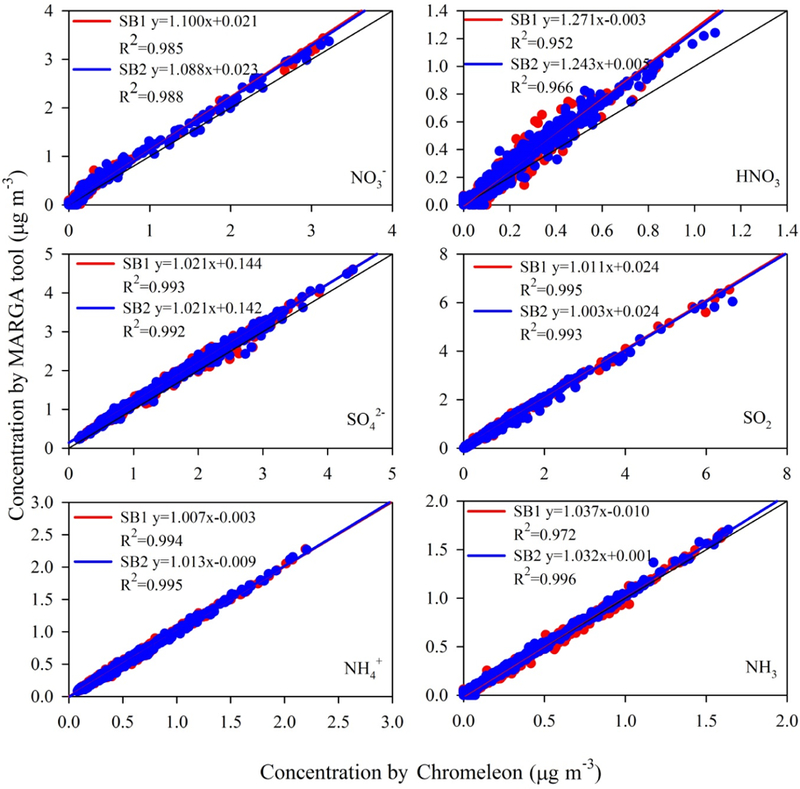
Comparison of concentrations of analytes monitored during fall of 2014 at Duke Forest as reported by MARGA tool and Chromeleon. Data points with misintegration issues by MARGA tool were excluded from this comparison. Data for individual sample boxes (SB1 and SB2) are shown.

Air concentrations derived from the MARGA and Chromeleon software approaches were compared by ordinary least-squares regression using Chromeleon as the reference (Fig. 1). Over the entire range of conditions, concentrations calculated using the MARGA tool were within 5 % (slopes, Fig. 1), on average, of those reported by Chromeleon for $SO_{4}^{2-}$, SO_2_, $NH_{4}^{+}$ and NH_3_. Very good agreement is observed for $NH_{4}^{+}$ and NH_3_, with slopes close to unity and intercepts near zero. As concentrations were below 2.5 µg m^−3^, potential disagreement resulting from differences in cation integration at higher concentrations (Sect. 3.1) was not observed. Although the accuracy of $NO_{3}^{-}$ was poorer, it was within 10 % overall. By contrast, HNO_3_ concentrations, which were mostly below 1.0 µg m^−3^, showed a positive bias of approximately 30 %. Correlation of HNO_3_ between the MARGA tool and Chromeleon also revealed a more scattered pattern compared to other analytes. The 30 % positive bias in MARGA HNO_3_ results is also observed for $NO_{3}^{-}$ concentrations below ≈ 1.0 µg m^−3^ (Fig. S6). Restricting the $NO_{3}^{-}$ regression comparison to lower concentrations results in slopes of ≈ 1.4 and ≈ 1.5 over concentration ranges of 0–0.5 and 0–0.25 µg m^−3^, respectively, with intercepts near zero; the disagreement increases at concentrations below 0.25 µg m^-3^. SO_2_ and $SO_{4}^{2-}$ results also show pos-itive bias in the MARGA results at lower concentrations, though not as large as observed for HNO_3_ and $NO_{3}^{-}$. For SO_2_, slopes of ≈ 1.1, 1.15 and 1.2 are observed over concentration ranges of 0–1.0, 0–0.5 and 0–0.25 µg m^−3^, respectively, with intercepts near zero. Agreement improves at concentrations above 1.0 µg m^−3^ as the slope approaches unity. Over the entire range of conditions, $SO_{4}^{2-}$ also shows good agreement, on average, though with a significant offset (0.14 µg m^−3^, Fig. 1). At lower concentrations (Fig. S6), a pattern of disagreement similar to SO_2_ emerges; over the range 0–1.0 µg m^−3^, a slope and intercept of 1.09 and 0.09 are observed, respectively. Similar discrepancy patterns were observed for $SO_{4}^{2-}$ and $NO_{3}^{-}$ when lower level external standards were tested. In contrast to anions, cation results showed consistently good agreement even at low concentrations.

The source of bias between the MARGA and Chromeleon results may result from several factors: (1) MARGA overestimation from incorrectly defined peak start and end points due to peak fronting and tailing; (2) incorrect baseline definition for smaller peaks (i.e., low observed HNO_3_ and $NO_{3}^{-}$ concentrations) as compared to larger peaks; or, perhaps the most likely explanation, (3) inability of the van Os et al. (1984) method used by the MARGA software to fully linearize the relationship between peak area and liquid concentration at low concentrations. As noted above, the method of van Os et al. (1984) for anion analysis with chemical suppression allows calculation of the sample concentration directly from the conductivity measurement. Van Os et al. (1984) concluded that relationships between the amount of sample injected and total peak area were linear over the range 2.0– 40.0 mg L^-1^. It was noted, however, that calculated concentrations at the 1.0 mg L^−1^ standard level, the lowest concentration tested, were slightly low for $NO_{3}^{-}$ and Cl^–^ and slightly high for $SO_{4}^{2-}$. Subsequently, the 1.0 mg L^−1^ standard level was not used in the final regression analysis used to test the linearity of the method. Accounting for differences in injection loop size between studies, the 1.0 mg L^−1^ level used by van Os et al. (1984) is a factor of 2 to 2.5 larger than the highest standard concentration tested in our study (Table S1) and a factor of 25 (SO_2_) to 125 (HNO_3_) larger than the corresponding average observed air concentrations (Table 2). It is possible that the method of van Os et al. (1984) systematically overpredicts anion concentrations at the lower concentrations observed in our study. This accuracy issue would not be controlled by the single-point Br internal standard (3680 µg L^−1^), which is within the linear response range of anion concentrations tested by van Os et al. (1984).

The $NO_{3}^{-}$ bias observed here may help to explain the results of previous studies. Five semi-continuous analyzers, which included an earlier version of a wet annular denuder/SJAC (Trebs et al., 2004, 2008) that predates the commercialized MARGA, were evaluated and intercompared by Weber et al. (2003) for measurements of $NO_{3}^{-}$ and $SO_{4}^{2-}$ in PM_2.5_ at the Atlanta EPA supersite. The earlier version MARGA analyzer showed a range of 25 to 34 % significantly higher $NO_{3}^{-}$ concentration as compared to a group mean of the five semi-continuous monitors evaluated while measured $SO_{4}^{2-}$ agreed well (within 10 %). This discrepancy was suspected to be a sampling artifact of $NO_{3}^{-}$ formed from NO_*x*_ in the MARGA particle steam collector, though there was a lack of correlation with measured NO_*x*_. Four instruments including a MARGA, an AMS, a denuder difference analyzer and an integrated nylon-filter-based IMPROVE sampler were evaluated by Allen et al. (2015) during the 2013 Southern Oxidant and Aerosol Study (SOAS) campaign for particulate $NO_{3}^{-}$. The MARGA measured much higher $NO_{3}^{-}$ concentrations than the other three analyzers at this southeastern US site, where $NO_{3}^{-}$ was mostly below 1.0 µg m^−3^ during the sampling period. Differences in inlet cyclone size cuts and cyclone efficiencies for supermicron particles may be partly responsible. However, these examples of significantly higher MARGA $NO_{3}^{-}$ relative to other methods, as well as the results of this study, warrant further investigation of potential chromatography related biases.

#### 3.2.2 MARGA precision

**Figure 2.**
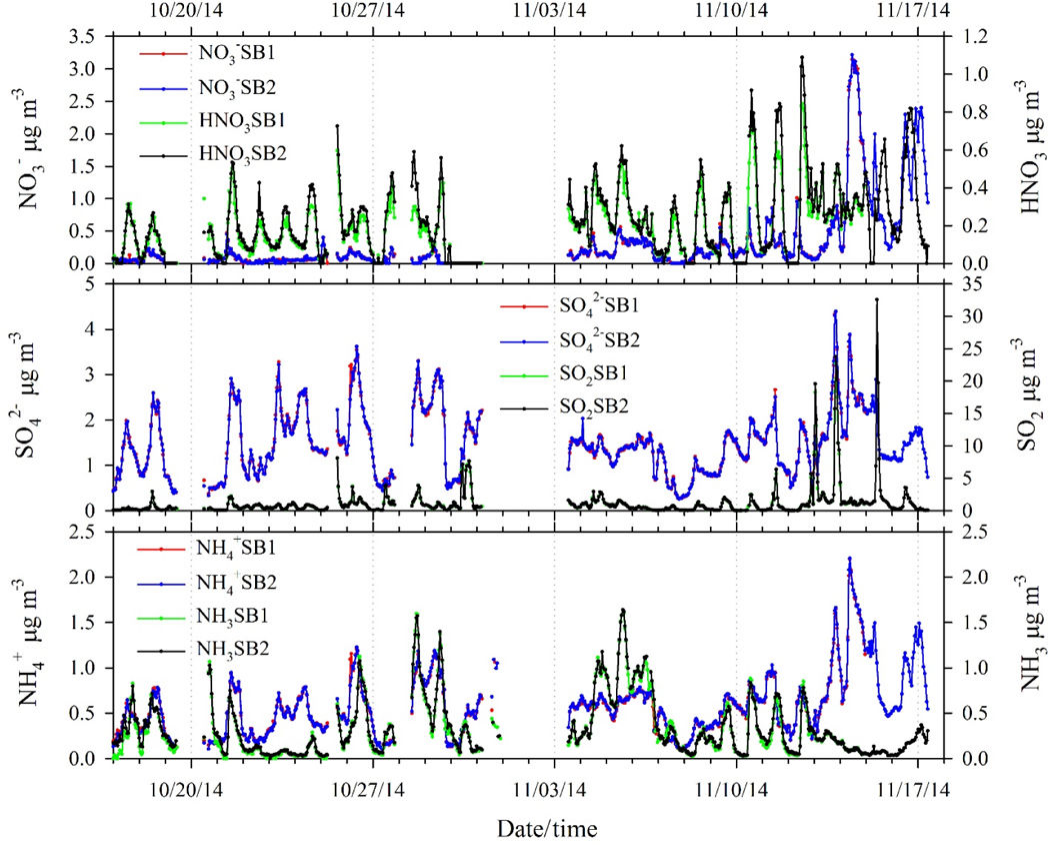
Time series of concentrations of particulate $NO_{3}^{-}$, $SO_{4}^{2-}$ and $NH_{4}^{+}$ and gas-phase HNO_3_, SO_2_ and NH_3_ by collocated MARGA sample boxes 1 (SB1) and 2 (SB2).

**Figure 3.**
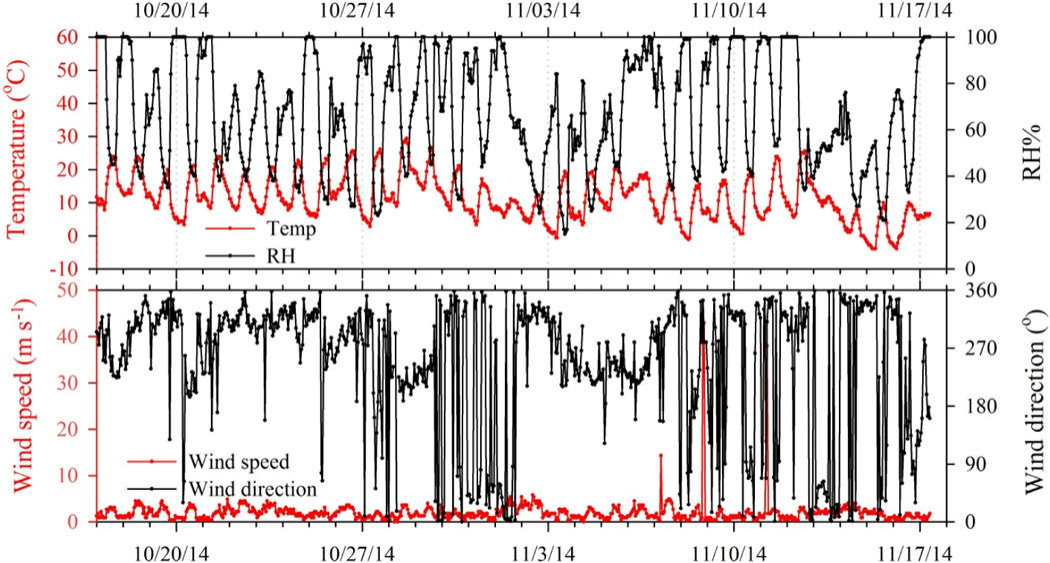
Hourly temperature, relative humidity, wind speed and wind direction during the fall 2014 field intensive.

Precision statistics (Table 2) were derived from orthogonal least-squares regression (Wolff et al., 2010) of concentrations from the two MARGA sample boxes operated in parallel (i.e., collocated). Orthogonal least-squares regression acknowledges uncertainty in both the *X* and *Y* variables (i.e., measurements from both sample boxes) and the standard deviation of the residuals of the regression is therefore a measure of the overall precision of the MARGA system. Concentrations of particulate $NO_{3}^{-}$, $SO_{4}^{2-}$, $NH_{4}^{+}$, gaseous SO_2_ and NH_3_ agree well between the sample boxes, with slopes within 5 % of unity and negligible intercepts (Table 2), indicating no significant systematic differences between the two sample boxes. The standard deviations (precision) and relative standard deviations (RSD, expressed as a percentage of the average air concentrations) of the regression residuals reported here (µg m^−3^) for $NO_{3}^{-}$, $SO_{4}^{2-}$, $NH_{4}^{+}$ and NH_3_ are similar (< 10 % RSD) to those reported by Rumsey and Walker (2016). The lower precision for SO_2_ reported here is most likely related to larger differences in concentration between sample boxes during periods of rapid concentration changes associated with the Arctic air episode (Figs. 2 and 3).

Relative to the other analytes, HNO_3_ showed a much more significant difference between the two sampler boxes (regression slope of 0.83). Additionally, HNO_3_ precision (15.8 % RSD) was much lower than observed for $NO_{3}^{-}$ aerosol (4.8 % RSD) at nearly identical average concentrations. These findings, in combination with the excellent agreement between sample boxes for $NO_{3}^{-}$, suggest that the HNO_3_ measurements were influenced by inlet rather than analytical issues. As indicated by the much higher Henry’s law coefficient of HNO_3_ relative to NH_3_ and SO_2;_ HNO_3_ is “sticky” and therefore more prone to inlet losses as well as reevaporation from inlet/tubing surfaces. Although the inlet cyclones used were coated with Teflon and the Teflon tubing connecting the cyclone to the WRD was very short (0.2 m), our results suggest differences in transmission efficiencies of the two inlets. Similar difficulties in sampling HNO_3_ have been reported previously for studies in which size selective inlets and/or significant lengths of sample tubing were used for MARGA sampling (Trebs et al., 2004; Rumsey et al., 2014; Allen et al., 2015). In our study, the length of inlet tubing between the cyclone and WRD was similar to the length of tubing (0.3 m) used by Rumsey and Walker (2016), the difference being that no size selective inlet was used by Rumsey and Walker. In their study, multiple collocation experiments showed much better agreement, on average, between the two sample boxes and better precision (5.8 % RSD), suggesting that the cyclone may be the primary source of disagreement between sample boxes in the current study. It is important to note, however, that concentrations of HNO_3_ observed in the current study were generally very low, averaging 0.19 µg m^−3^ over the study period. Such low concentrations contribute to greater relative variability between sample boxes. Our results reemphasize the requirement of low-affinity tubing and inlets with respect to both materials and surfaces/lengths for HNO_3_ sampling.

#### 3.2.3 Temporal patterns of gas and particle concentrations

Figure 2 shows time series of hourly gas-phase concentrations of HNO_3_, SO_2_ and NH_3_ and particle-phase $NO_{3}^{-}$, $SO_{4}^{2-}$ and $NH_{4}^{+}$ (as local time; EDT). From mid-October to mid-November, meteorological conditions were mild and humid (Fig. 3), which is typical of fall in the southeastern US. However, an Arctic outbreak of cold air impacted the site from 13 to 17 November, accompanied by much lower temperature and relative humidity. Wind speed was typical of the site, averaging 2 m s^−1^. The prevailing wind directions were north-west and southwest before the cold air period and northerly during the dry and cold period.

**Figure 4.**
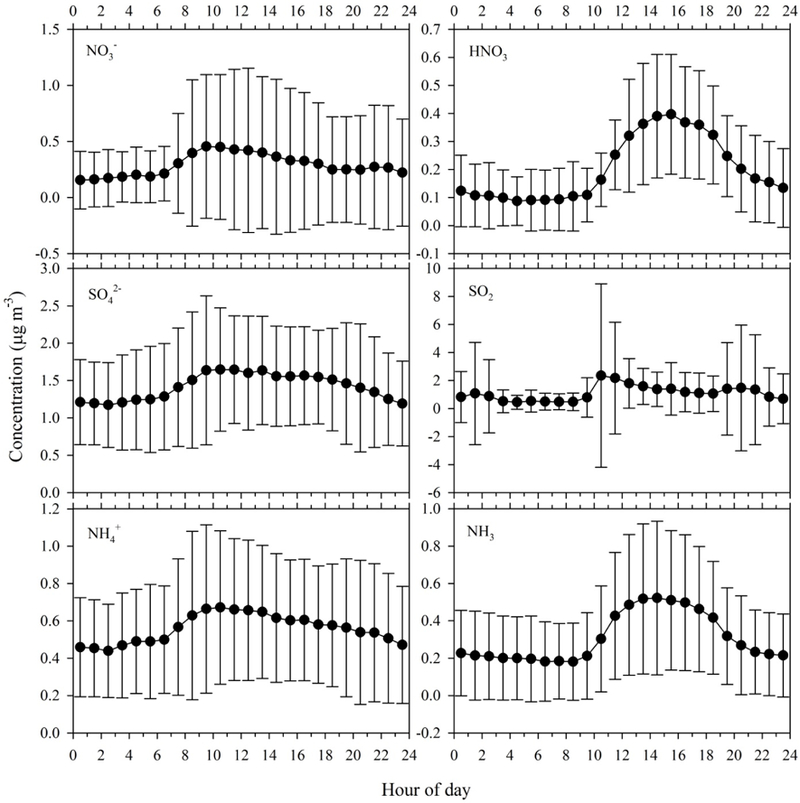
Diurnal profiles of particulate $NO_{3}^{-}$, $SO_{4}^{2-}$ and $NH_{4}^{+}$, gas-phase HNO_3_, SO_2_ and NH_3_ during the fall 2014 field intensive. Data points represent average concentrations, while error bars represent 1 standard deviation.

Figure 4 shows the diurnal pattern of gas and particle concentrations. Only days with hourly data coverage greater than 65 % were used for calculating diurnal profiles (*N* = 26). $NH_{4}^{+}$ and $SO_{4}^{2-}$ exhibited a single mode pattern with a peak around 09:00–11:00 local time. $NO_{3}^{-}$ showed a similar peak in the morning and a smaller peak at 21:00–23:00. Morning peaks most likely represent the downward mixing of aerosols from aloft when the nocturnal boundary layer breaks down. The second peak of $NO_{3}^{-}$ at night may be related to night-time $NO_{3}^{-}$ radical chemistry (Finlayson-Pitts and Pitts, 2000; Seinfeld and Pandis, 2006) leading to formation of particulate $NO_{3}^{-}$. The mid-afternoon (14:00–15:00) peak in gas-phase HNO_3_ results from photochemical processing of NO_*x*_. NH_3_ showed a much broader afternoon peak, which may reflect local emissions from natural sources during warmer afternoon periods. The diurnal pattern of SO_2_ showed a pronounced peak around 10:00–11:00 and two less pronounced peaks at 20:00 and 01:00, respectively. This pattern may reflect the competition between emission and dry deposition, as well as boundary layer dynamics: higher emissions during the day versus slower dry deposition rates and shallower boundary layer at night. The diurnal pattern is also affected by the large SO_2_ spikes observed during the Arctic air mass period, presumably associated with increased emissions resulting from greater energy demand.

**Figure 5.**
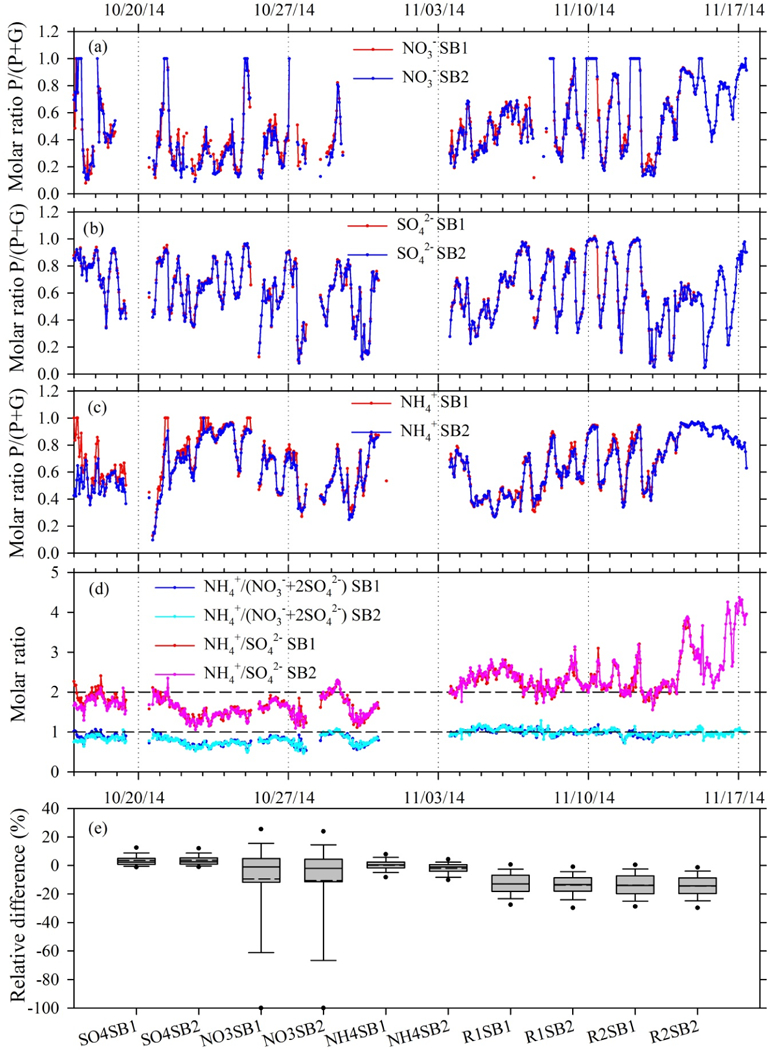
Partitioning molar ratios of (a) $NO_{3}^{-}$, (b) $SO_{4}^{2-}$ and (c) $NH_{4}^{+}$ episodes, with the majority in particle phase, calculated as particle / (particle+gas). (d) Molar ratios (R1and R2) of particulate $NO_{3}^{-}$, $SO_{4}^{2-}$ and $NH_{4}^{+}$ to determine particle neutralization state and acidity. (e) Relative difference of parti-tioning molar ratios of $NO_{3}^{-}$, $SO_{4}^{2-}$ and $NH_{4}^{+}$ in particle phase as well as particle neutralization state indicators R1 and R2 by Chromeleon and MARGA tool. Negative values indicate a lower ratio calculated by the MARGA tool (i.e., positive bias in concentrations calculated by MARGA tool). Solid and dashed lines inside the boxes represent median and mean, respectively. Top and bottom boxes represent 75th and 25th percentiles. Whiskers represent 90th and 10th percentiles. Dots represent 95th and 5th percentiles. SB1 and SB2 indicate collocated MARGA sample boxes 1 and 2, respectively.

Gas–particle partitioning presented as fraction in the particle phase is shown in Fig. 5. In order to examine the aerosol neutralization state, chemical composition ratios were calculated as

(1) $$R1=\frac{NH_{4}^{+}}{SO_{4}^{2-}},$$

(2) $$R2=\frac{NH_{4}^{+}}{NO_{3}^{-}+2\times SO_{4}^{2-}},$$

where ratios R1 and R2 are molar concentration based. R1 = 2 reflects an aerosol entirely composed of (NH_4_)_2_SO_4_, which is the fully neutralized state of $SO_{4}^{2-}$. R1 > 2 indicates the presence of NH_4_NO_3_ in addition to (NH_4_/_2_SO_4_, while R1 < 2 signifies a state of deficit indicative of an acidic aerosol. Moreover, a ratio of R2 = 1 indicates a fully neutralized aerosol containing NH_4_NO_3_ and (NH_4_)_2_SO_4;_ while R2 > 1 represents as condition of excess $NH_{4}^{+}$. A value of R2 < 1 suggests acidic aerosol comprising NH_4_NO_3_ and a combination of NH_4_HSO_4_ and (NH_4_)_2_SO_4_ or, alternatively, NO_3_ associated with supermicron particles from aged sea salt or crustal materials (Allen et al., 2015).

Two distinct periods of contrasting aerosol composition were observed (Fig. 5d). With R1 mostly less than 2 and R2 less than or close to 1, aerosol measured during October primarily comprised NH_4_HSO_4_ and (NH_4_/_2_SO_4_. When R1 approached 1 for three short episodes in October, particles most likely existed solely as NH_4_HSO_4_. The observed acidity most likely suppressed $NO_{3}^{-}$ partitioning and formation, which is reflected by a significant decrease in the molar ratio of $NO_{3}^{-}$ in aerosol phase to as low as 0.1–0.2 (Fig. 5a). Limited aerosol $NO_{3}^{-}$ formation was also reported by Allen et al. (2015) at a southeastern US site where aerosol was acidic. By contrast, R1 was mostly above 2 in November, indicating the presence of $NO_{3}^{-}$. From 13 to 17 November, R1 reached as high as 4. Nevertheless, R2 was generally close to 1 during November, indicating an aerosol comprised of NH_4_NO_3_ and (NH_4_/_2_SO_4_. In contrast to the $SO_{4}^{2-}$ -dominated October period, $NO_{3}^{-}$ was a much greater contributor to inorganic aerosol in November; molar concentrations of NH_4_NO_3_ even surpassed (NH_4_/_2_SO_4_ when R1 reached 4 during the cold air event. It should be noted that only acidity from inorganic species was examined in this study and the ion balance could be further affected if organic acids were present and taken into account.

As noted above and illustrated in Fig. S6, a positive bias in $NO_{3}^{-}$ and $SO_{4}^{2-}$ resulting from peak integration and processing with the MARGA tool is observed for air concentrations below ∼ 1.0 µg m^−3^. Our field study provides an opportunity to quantify the impact of these errors over a range of chemical and meteorological conditions. For this analysis, the difference between hourly concentrations determined by the MARGA versus Chromeleon software was calculated as a percent relative to the Chromeleon result (i.e., 100 % (MARGA–Chromeleon)/Chromeleon). Overall statistics of the hourly relative differences are summarized in Fig. 5e, including differences in phase partitioning (i.e., molar ratios calculated as particle = (particle + gas)) and neutralization state (R1 and R2). As expected, differences in the $NH_{4}^{+}$ = NH_3_ partitioning ratio are near zero because no bias was observed between Chromeleon-and MARGA-derived concentrations of NH_3_ and $NH_{4}^{+}$. Average and median differences in the $SO_{4}^{2-}$ = SO_2_ partitioning ratio were similarly small, which is expected given that average $SO_{4}^{2-}$ and SO_2_ concentrations were 1.41 and 0.98 µg m^−3^, respectively (Table 2). These concentrations are above the level at which biases between MARGA and Chromeleon become significant. Mean and median differences in the $NO_{3}^{-}$ = HNO_3_ partitioning ratio were ≈ −10 and 1.5 %, respectively, indicating a smaller ratio calculated with the MARGA software. As shown in Fig. 5e, the $NO_{3}^{-}$ = HNO_3_ partitioning ratio exhibits much larger hourly variability relative to the other analytes, reflecting a combination of larger concentration bias and random error associated with integration of very small peaks. The average relative difference in R1 was ≈ − 13 %, resulting from the combination of a constant offset and concentration-dependent difference between MARGA versus Chromeleon $SO_{4}^{2-}$ results (Sect. 3.2.1). Differences in R1 increase nonlinearly with decreasing $SO_{4}^{2-}$ concentration, reaching ≈ −25 % at 0.5 µg $SO_{4}^{2-}$ m^−3^. The average relative difference in R2 was ≈ −14 %, also exhibiting larger differences at lower concentrations. Following the propagation of error in R2, differences are primarily driven by much higher absolute concentrations of $SO_{4}^{2-}$ relative to $NO_{3}^{-}$. Though absolute differences are larger for $NO_{3}^{-}$ concentrations, low concentrations result in a lesser contribution to the overall difference in R2 between the MARGA and Chromeleon methods.

#### 3.2.4 Arctic event

As noted above, an Arctic outbreak of cold air impacted the site from 13 to 17 November. The average temperature dropped from 12.9 to 4.5 ^°^C during this period, with a minimum of 3.9 ^°^C, which is well below normal for this site. RH ranged from 21 to 77 % during the cold air event. Total concentrations of gases plus particles were ≈ 2 × higher during the cold Arctic event for NH_3_,, $SO_{4}^{2-}$ and SO_2_; while for $NO_{3}^{-}$ and HNO_3_, a factor of 5 difference was observed (summary shown in Table 3). Though air was drier during the Arctic event, temperatures were cold enough to drive partitioning of gas-phase inorganic compounds towards the particle phase. In addition to elevated $NO_{3}^{-}$ concentrations, three distinct episodes of SO_2_ occurred, with a maximum concentration of 32.56 µg m^−3^ (Fig. 2). Back trajectory analysis (Fig. S7) suggests that these SO_2_ events reflect transport of emissions from power plants and other point sources in the Midwest (see facility SO_2_ emission inventories Fig. S8). SO_2_ from more local sources during the extremely dry and cold Arctic air conditions might also have contributed to the observed SO_2_ spikes.

**Figure 6.**
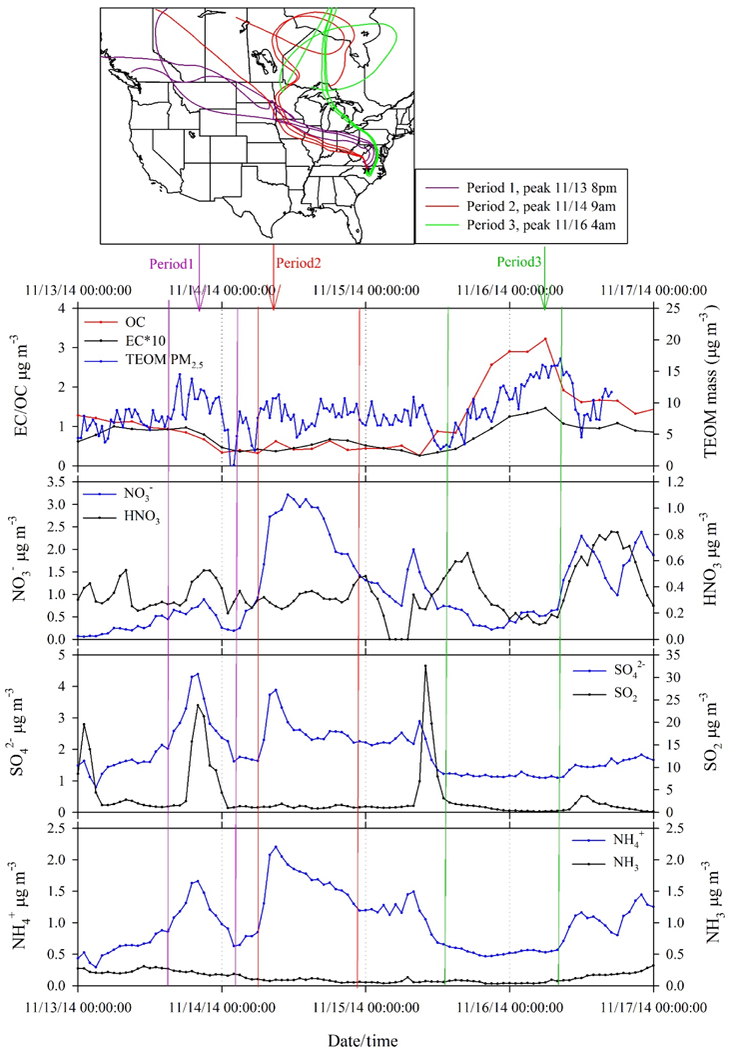
High-concentration periods observed during mid-November 2014. Period 1: highest $SO_{4}^{2-}$ ; Period 2: highest $NH_{4}^{+}$ and $NO_{3}^{-}$ ; Period 3: highest OC. Corresponding back trajectories (arrival at 500AGL, backwards for 168 h) of individual period peaks (±2 h) are also presented.

Gas and particle chemistry during the 13 to 17 November period, including TEOM PM_2.5_ mass and EC / OC concentrations, are examined in more detail in Fig. 6. This fourday period represents the highest concentrations of $SO_{4}^{2-}$, $NH_{4}^{+}$, $NO_{3}^{-}$ and OC concentrations, as well as lowest temperature, observed during the study. However, total PM_2.5_ mass showed less variability than the other species. Summaries of concentrations of gaseous and particulate species are presented in Table 3 during and outside of the cold air event. In order to better examine the Arctic air mass intrusion, three subperiods were selected, featuring a high $SO_{4}^{2-}$ episode; high $NH_{4}^{+}$ and $NO_{3}^{-}$ episode; and a high OC episode (individual periods are marked and color coded in Fig. 6). Inorganic components in particles demonstrated a pattern of high concentrations for periods 1 and 2, while less so during period 3. Particulate organic composition as represented by OC showed an opposite pattern, peaking in period 3. Differences in time resolved concentrations of inorganic and organic species illustrate different emission sources for inorganic and organic particulate pollutants. Back trajectories associated with the three episodes are presented in Fig. 6. For inorganic episodes 1 and 2, air masses originated from the Arctic and passed through the US Midwest and Ohio River valley where emissions of inorganic aerosol precursors, SO_2_ and NO_*x*_, from power plants and heavy industries were encountered. Gas-phase NH_3_ concentrations are very low during these episodes, with the majority of NH_*x*_ in the particle phase. By contrast, trajectories associated with the high OC episode (period 3) suggest more of a northeastern origin and perhaps a greater influence of residential wood burning associated with cold temperatures. During periods 1 and 2, inorganic compounds contributed the majority of PM_2.5_ mass. The estimated sum of inorganics including $SO_{4}^{2-}$, $NO_{3}^{-}$ and $NH_{4}^{+}$ accounted for 61 ± 31 and 83 ± 24 %, respectively, of the PM_2.5_ mass for periods 1 and 2. In contrast, inorganic compounds only accounted for 22 ± 11 % of PM_2.5_ mass during period 3.

## 4 Summary and conclusions

The MARGA is a state-of-art instrument that measures near-real-time water-soluble particulate species and their gaseous precursors. The current commercial version of the MARGA incorporates a continuous internal standard (LiBr) to verify and calibrate instrument response for automated data generation and reporting. Close examination of MARGA chromatograms revealed a number of issues, including misidentification and misintegration of analyte peaks. Peak integration across similar chromatograms was found to be inconsistent with the MARGA software shifting between integration options “drop perpendicular” and “valley to valley” among samples. In addition, $NO_{3}^{-}$ peaks were rather frequently discovered as not integrated or identified; $SO_{4}^{2-}$ peaks were found to have an incorrectly defined baseline due to peak fronting and tailing. Adjustment of individual peak integrations was found to be difficult and inefficient with features provided by MARGA tool software. Hence, an alternative integration software, Chromeleon by Thermo Scientific Dionex, was used to reprocess the raw chromatograms. A custom Java script was developed to incorporate MARGA raw conductivity data into Chromeleon for reprocessing.

Though a number of chromatography issues with the MARGA commercial software were identified, a relatively small percentage (6.2 %) of data, overall, were invalidated due to peak misintegration issues during the 1-month field study described here. $NO_{3}^{-}$ peaks appeared to be the analyte most affected and higher rates of data invalidation may be expected where $NO_{3}^{-}$ concentrations are typically low. The additional flexibility and consistency of Chromeleon in integrating small peaks results in lower method detection limits relative to the MARGA chromatography software. Very good agreement between the two chromatography methods was observed for cations across the range of observed ambient concentrations and for anions at concentrations above ∼ 1 µg m^−3^. At ambient concentrations below ∼ 1 µg m^−3^, however, concentrations determined using the MARGA software are biased +30 and +10 % for $NO_{3}^{-}$ and $SO_{4}^{2-}$, respectively, compared to concentrations determined using the alternative chromatography procedure. Differences between the two methods increase at lower concentrations. Over the range of conditions observed in our field study, the bias in $NO_{3}^{-}$ produces nontrivial errors in average $NO_{3}^{-}$ concentrations and metrics of particle acidity. While the cause of this bias is unclear, we make the following recommendations for controlling accuracy:

– Do not rely solely on the LiBr internal standard to ensure accuracy of the chromatographic analysis.
– Calibrate with multipoint curves using external liquid standards.
– Use a range of external standards appropriate for expected ambient concentration levels and for resolving potential nonlinearity in response at low concentrations.

During the field campaign, the MARGA captured rapid compositional changes in PM_2.5;_ including changes in neutralization state. A particularly high $NO_{3}^{-}$ episode associated with Arctic air mass intrusion and transport of pollutants from sources in the Midwest US was observed. Our field study further demonstrates the usefulness of the MARGA system for characterizing the temporal characteristics of the sulfate–nitrate–ammonium system associated with changes in local (i.e., diurnal) and synoptic-scale interactions between meteorology, emissions and aerosol processing.

## Supplementary Material

Supplement1

## Figures and Tables

**Table 1.** Method detection limits (MDL) for chromatograms processed by MARGA tool and reintegrated by Chromeleon.

	Chromeleon		MARGA tool	
	MDL (µg m^‒3^)	No.of samples	MDL (µg m^‒3^)	No.of samples
$NH_{4}^{+}$	0.02	78	0.04	78
NH_3_	0.02	78	0.04	78
$SO_{4}^{2-}$	0.08	80	0.13	76
SO_2_	0.05	80	0.08	76
$NO_{3}^{-}$	0.08	80	0.14	76
HNO_3_	0.08	80	0.14	76

**Table 2.** Comparison between MARGA sample boxes 1 and 2 for particulate $NO_{3}^{-}$, $SO_{4}^{2-}$ and $NH_{4}^{+}$ and gas-phase HNO_3_, SO_2_ and NH_3_ by orthogonal least-squares regression. *N* is number of observations, *C*_average_ is average air concentration,, σΔC is the standard deviation of the orthogonal least-squares residuals (i.e., detection limit; DL), σΔC/C_avg_ is the precision estimate, and *C*_max_ and *C*_min_ are the maximum and minimum air concentrations, respectively. Percentage of observations below the DL is also included.

	Slope	Intercept	$\sigma_{\Delta C}$ $\mu {\text{g m}}^{-3}$	*N*	^*C*^average $\mu {\text{g m}}^{-3}$	^*C*^max $\mu {\text{g m}}^{-3}$	^*C*^min $\mu {\text{g m}}^{-3}$	${\sigma_{\Delta C}/C}_{\mathrm{avg}}$ %	< DL %
$NH_{4}^{+}$	0.98	0.01	0.02	616	0.52	2.20	0.10	4	0
NH_3_	1.02	−0.03	0.03	614	0.33	1.62	0	9	5
$SO_{4}^{2-}$	0.99	0.01	0.05	602	1.41	4.39	0.17	4	0
SO_2_	0.96	0.02	0.15	603	0.98	23.26	0.01	15	27
$NO_{3}^{-}$	1.00	0.00	0.01	602	0.21	3.18	0	5	17
HNO_3_	0.83	0.01	0.03	603	0.19	0.97	0	16	20

**Table 3.** Summary of concentrations (µg m^−3^) of aerosol and precursor gases during and outside of cold air mass periods.

	Cold event			Non-cold event		
	Average	Median	Max	Average	Median	Max
NH_3_	0.12	0.09	0.29	0.35	0.24	1.62
HNO_3_	0.35	0.30	0.82	0.17	0.13	0.97
SO_2_	3.22	1.32	32.6	0.73	0.42	8.09
$NH_{4}^{+}$	0.99	0.88	2.20	0.48	0.45	1.21
$NO_{3}^{-}$	1.07	0.72	3.18	0.13	0.09	0.98
$SO_{4}^{2-}$	1.93	1.66	4.39	1.33	1.29	3.58
Temperature	4.54	5.00	13.9	12.9	12.2	29.4
RH %	50	51	77	70	71	100
