# Supplementary material for: Chromatography related performance of the Monitor for AeRosols and GAses in ambient air (MARGA): laboratory and field-based evaluation
Source: Atmos Meas Tech. Author manuscript; Available in PMC 2018 Oct 24. (PMC6192433; doi:10.5194/amt-10-3893-2017)
Supplement: Supplement1 [file NIHMS943800-supplement-Supplement1.pdf]

Supplement of Atmos. Meas. Tech., 10, 3893–3908, 2017
https://doi.org/10.5194/amt-10-3893-2017-supplement
© Author(s) 2017. This work is distributed under
the Creative Commons Attribution 3.0 License.

Atmospheric Measurement Techniques
Open Access

Supplement of

Chromatography related performance of the Monitor for AeRosols and GAses in ambient air (MARGA): laboratory and field-based evaluation

Xi Chen et al.

Correspondence to: John T. Walker (walker.johnt@epa.gov)

The copyright of individual parts of the supplement might differ from the CC BY 3.0 License.

Table S1. Concentrations (µg/L) of external liquid standards and certified accuracy check standard.

	Cl^-	NO_3^-	SO_4^{2-}	Na^+	NH_4^+	K^+	Mg^{2+}	Ca^{2+}
Level 1	3.28	2.15	2.07	0.42	2.06	0.35	0.21	0.24
Level 2	6.55	4.31	4.15	0.84	4.12	0.70	0.42	0.47
Level 3	16.39	10.77	10.37	2.09	10.30	1.74	1.05	1.19
Level 4	32.78	21.54	20.75	4.18	20.60	3.49	2.10	2.37
Level 5	65.55	43.07	41.50	8.36	41.20	6.98	4.20	4.74
Level 6	163.87	107.68	103.74	20.90	103.01	17.44	10.48	11.86
Level 7	327.74	215.36	207.48	41.80	206.02	34.88	20.95	23.72
Level 8	655.48	430.72	414.96	83.59	412.04	69.75	41.91	47.43
Check std	25.00	25.00	25.00	15.00	15.00	25.00	20.00	20.00

Table S2. Peak areas of internal standard LiBr as integrated by MARGA tool and Chromeleon over different external standard levels.

	MARGA tool		Chromeleon		Difference %		Average difference %	
	Br^- area	Li^+ area	Br^- area	Li^+ area	Br^-	Li^+	Br^-	Li^+
level 1	207.86	437.51	174.78	374.34	19	17	18	17
	214.16	444.26	183.06	382.26	17	16		
	209.94	449.25	178.38	379.98	18	18		
level 2	208.63	448.56	175.68	381.90	19	17	17	16
	207.29	446.07	177.42	381.18	17	17		
	207.36	439.45	179.16	388.08	16	13		
level 3	205.33	456.92	175.44	391.56	17	17	17	16
	206.85	439.84	176.10	378.06	17	16		
	207.14	450.44	174.90	387.60	18	16		
level 4	209.18	443.28	178.68	381.96	17	16	17	17
	206.42	450.00	177.00	382.26	17	18		
	206.28	465.22	176.82	397.14	17	17		
level 5	207.80	452.03	177.72	388.74	17	16	17	16
	208.56	444.99	178.86	383.70	17	16		
	204.07	442.72	174.30	383.28	17	16		
level 6	207.06	447.54	177.42	386.40	17	16	17	15
	207.88	440.05	178.08	384.30	17	15		
	206.26	444.83	177.06	388.56	16	14		
level 7	212.62	432.12	181.14	381.30	17	13	17	13
	205.94	442.50	176.64	389.88	17	13		
	207.03	448.96	177.78	398.40	16	13		
level 8	207.31	428.00	177.96	388.92	16	10	16	11
	208.51	426.98	182.04	383.40	15	11		
	206.86	435.68	177.18	390.36	17	12		

Table S3. Fraction of data points invalidated due to mis-identification and mis-integration by MARGA tool for sampling at Duke Forest during fall 2014.

	NO_3^-	SO_4^{2-}	NH_4^+	HNO_3	SO_2	NH_3
% Invalid	3.5	0.2	0.5	6.2	0.1	0.8
Total #	1271	1271	1300	1305	1305	1302

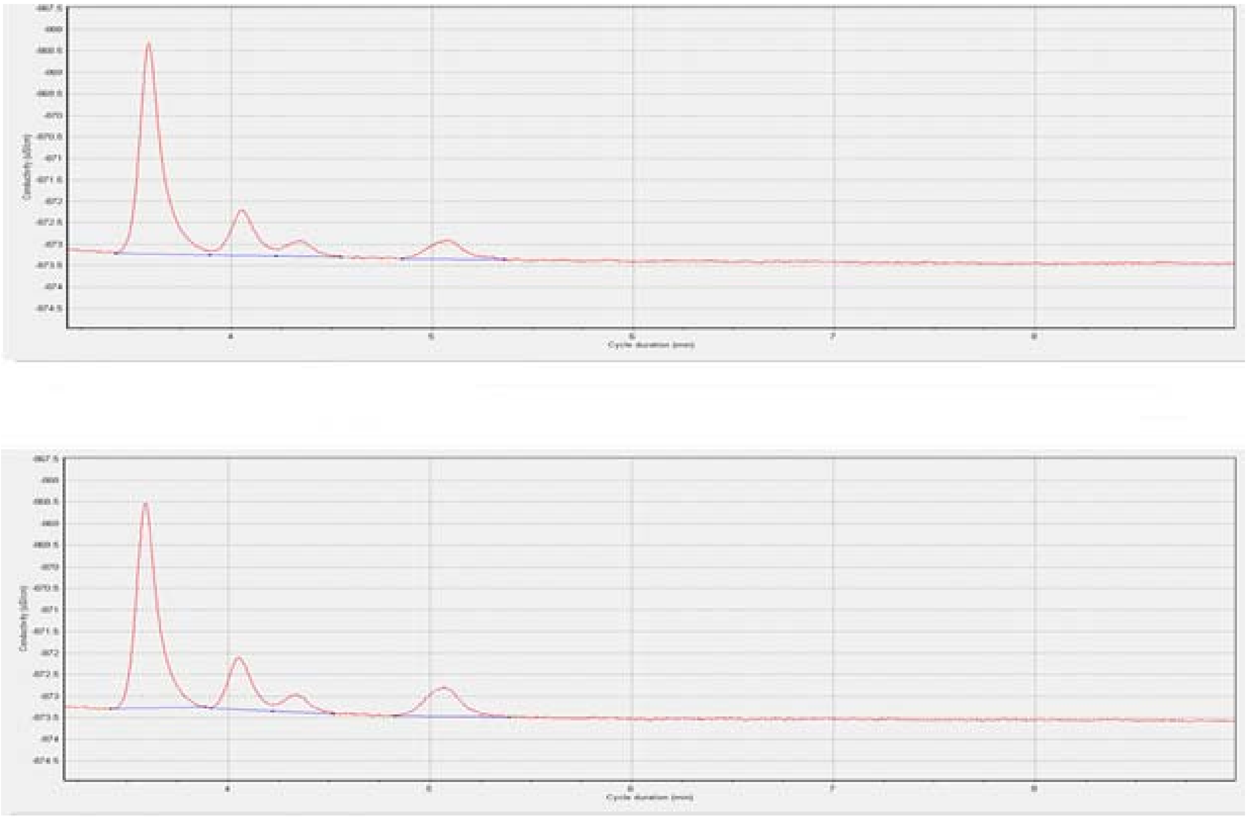

Figure S1. Chromatograms integrated by MARGA tool; top figure shows integration option of "drop perpendicular" applied to Li^+, Na^+ and NH_4^+ peaks; while the bottom figure shows option "valley to valley" applied to a parallel chromatogram (note MARGA tool only shows a dot representing peak start or end point; from left to right, the peaks shown should be Li^+, Na^+, NH_4^+ and K^+).

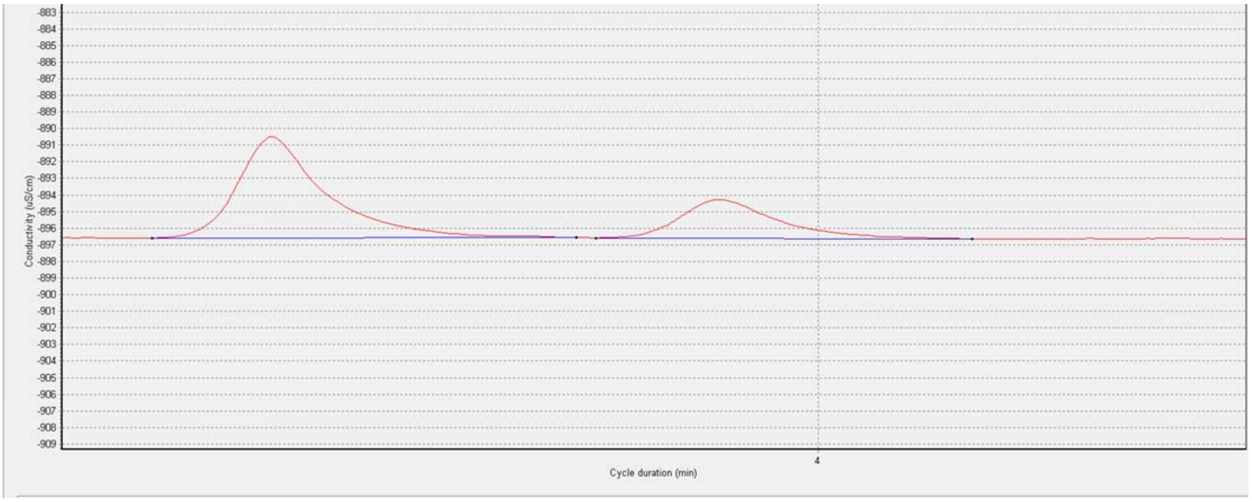

Figure S2. Chromatogram integrated by MARGA tool showing NH_4^+ peak mis-identified as Na^+(from left to right, the peaks shown should be Li^+ and NH_4^+).

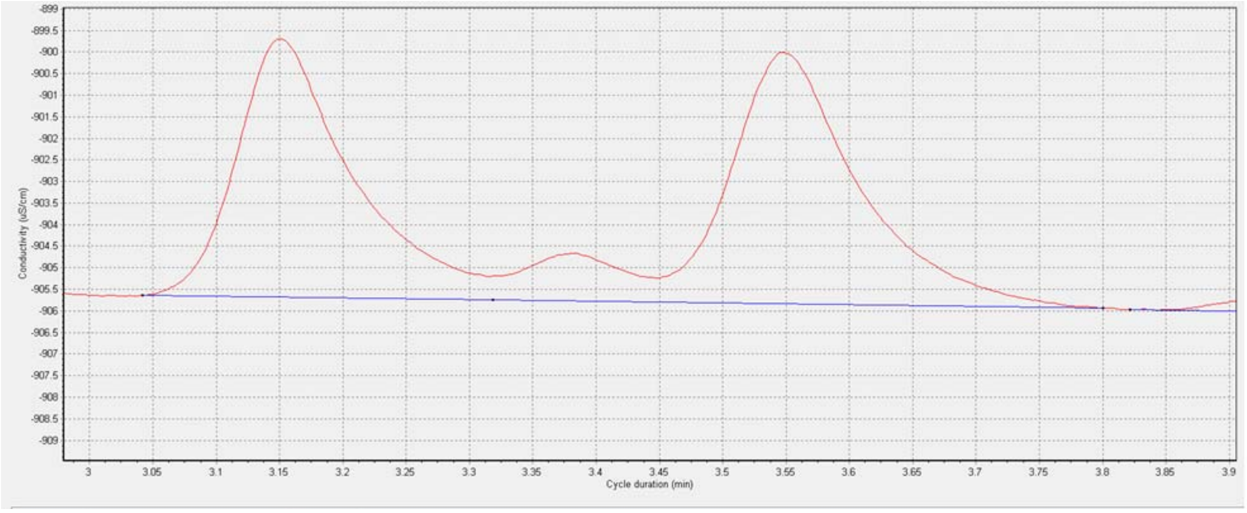

Figure S3. Chromatogram integrated by MARGA tool showing mis-identification of NH_4^+ and Na^+ peaks together as a single NH_4^+ peak(note MARGA tool only shows a dot representing peak start or end point; from left to right, the peaks shown should be Li^+ ,Na^+ and NH_4^+).

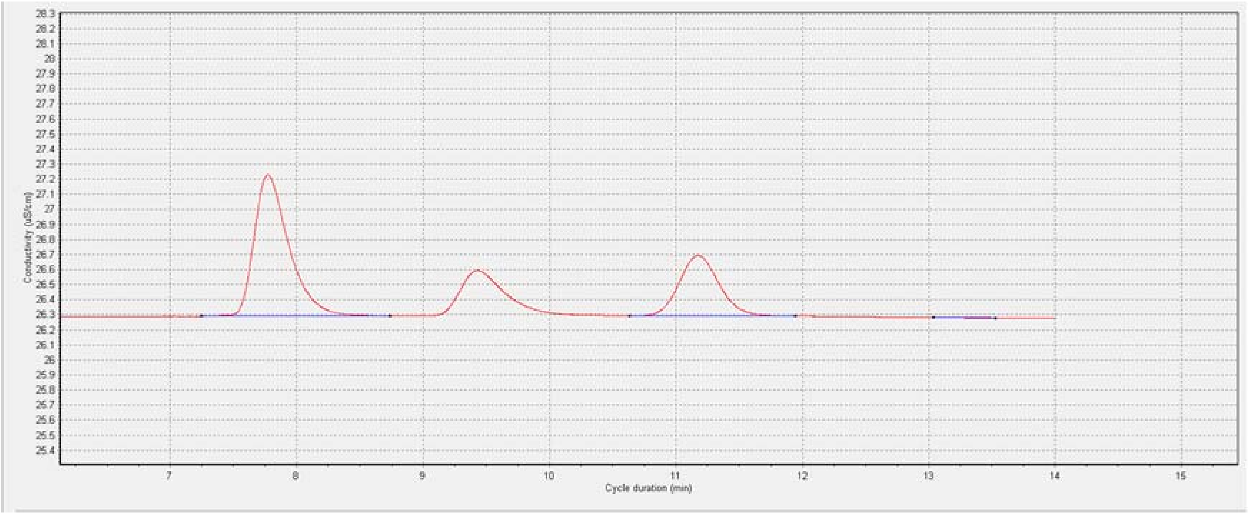

Figure S4. Chromatogram integrated by MARGA tool showing NO_3^- peak not integrated or identified (from left to right, the peaks shown should be Br^-, NO_3^- and SO_4^{2-}).

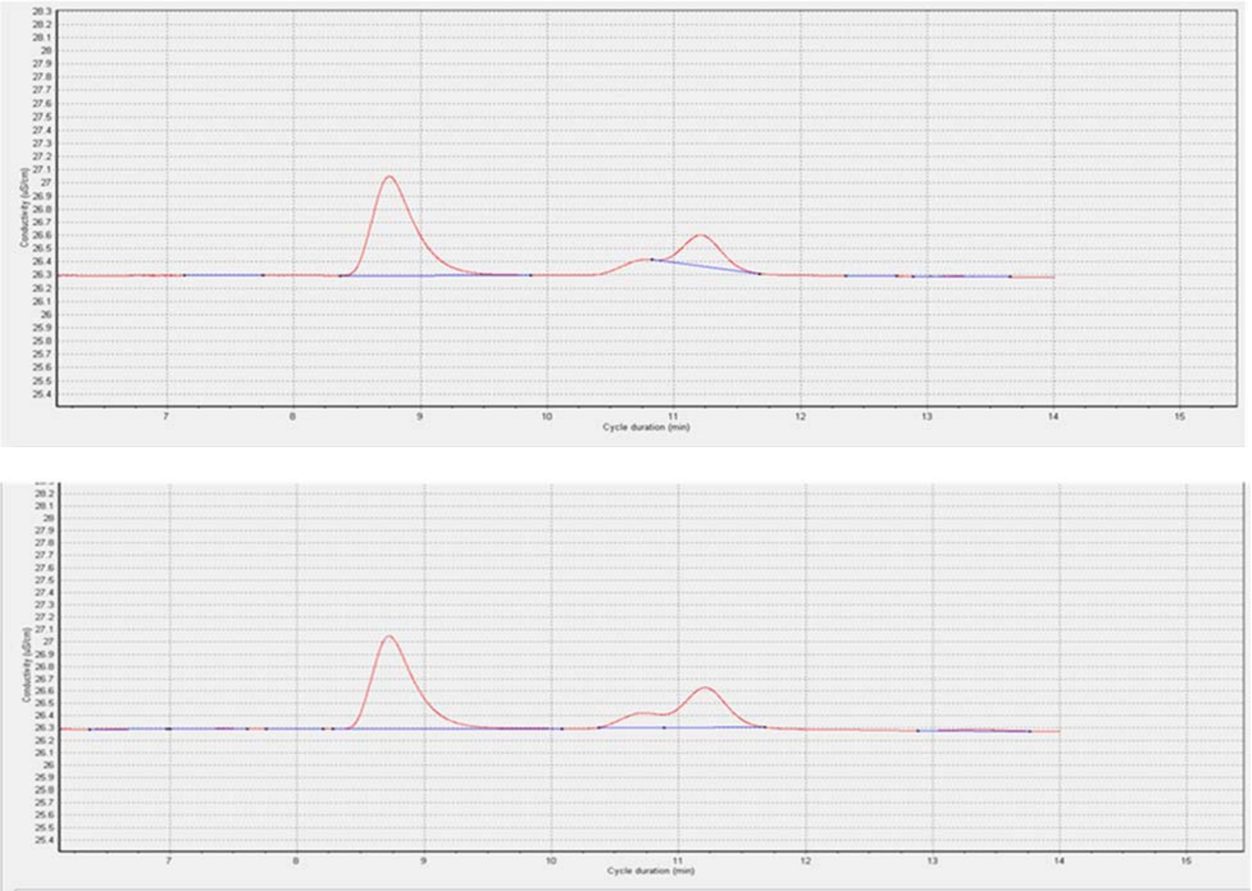

Figure S5. Chromatograms integrated by MARGA tool. Top figure shows “valley to valley” integration of SO_4^{2-} peak while NO_3^- peak was not identified and integrated for a sample by sample box 1. Bottom figure shows integration option “drop perpendicular” applied to a parallel sample of the same hour by sample box 2 in which both SO_4^{2-} and NO_3^- peaks were identified and integrated (from left to right, the peaks shown should be Br^-, NO_3^- and SO_4^{2-}).

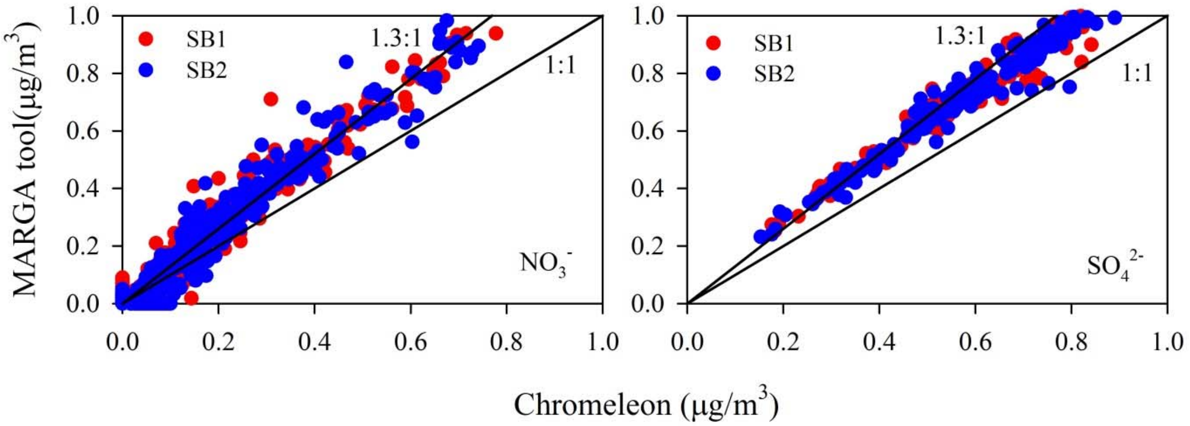

Figure S6. Comparison of NO_3^- and SO_4^{2-} during fall 2014 field intensive at Duke Forest as reported by MARGA tool and Chromeleon for concentration regions lower than 1.0µg/m^3. Data mis-integrated by the MARGA tool were excluded from this comparison (SB: sample box, lines 1.3:1 and 1:1 are shown as guide).

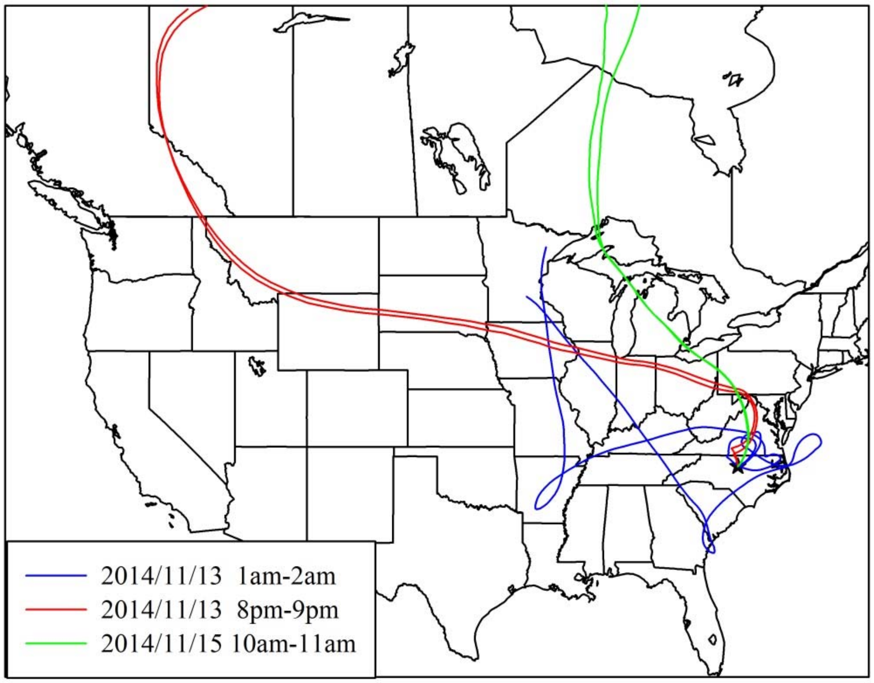

Figure S7. Corresponding back trajectories (arrival at 500AGL, backwards for 168hrs) of 3 spike peaks (±1hr) of observed SO_2 concentrations.

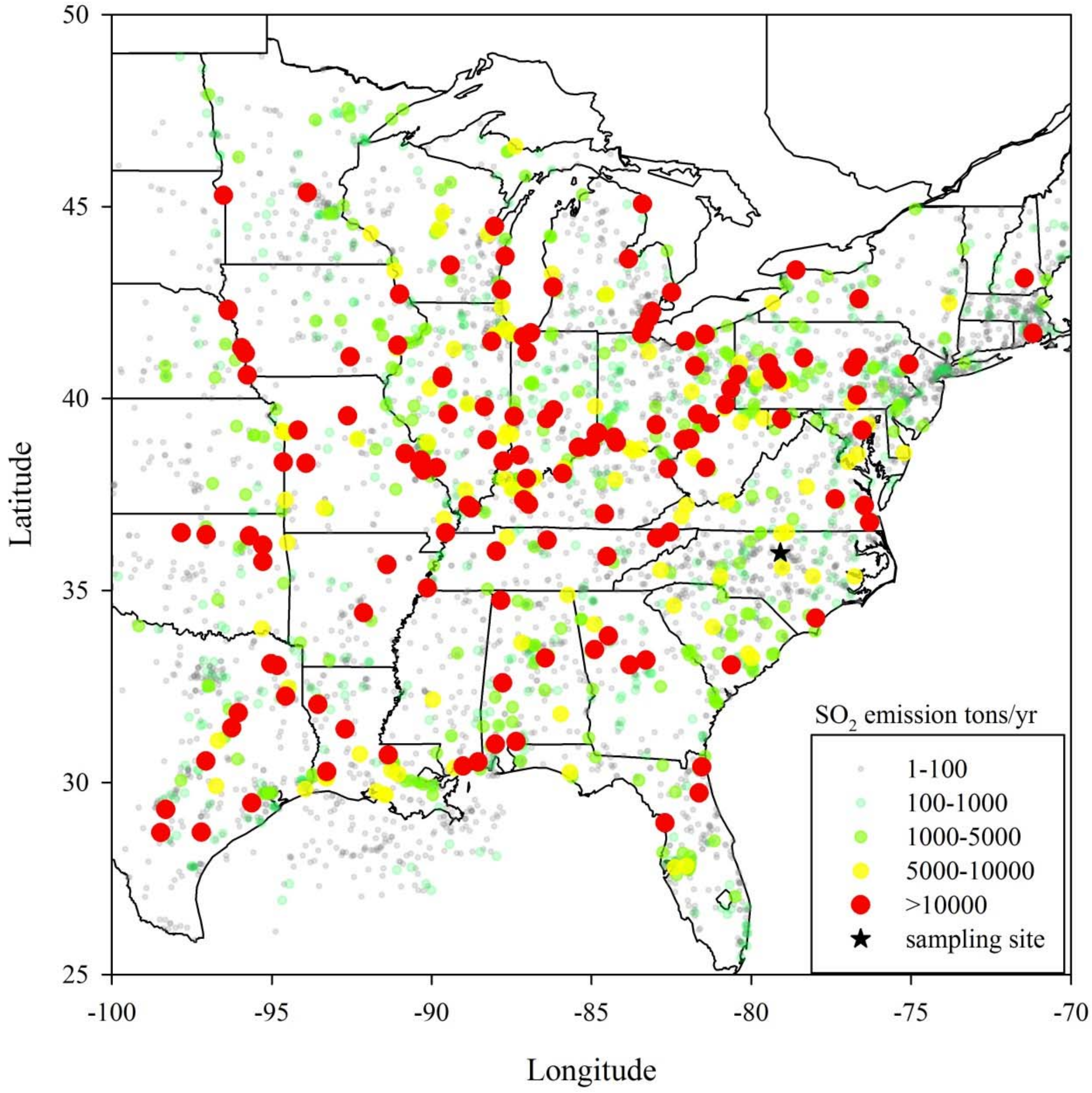

Figure S8. SO_2 Emission inventory map covering mid and eastern US from point sources 2011.
